# Differences in Susceptibility to Cyprinid Herpesvirus 3 (CyHV-3) Infection Among Carp (*Cyprinus carpio* L.) Strains and Hybrids

**DOI:** 10.3390/v18040432

**Published:** 2026-04-02

**Authors:** Xiaona Jiang, Zhenguo Song, Chitao Li, Xuesong Hu, Yanlong Ge, Lei Cheng, Xiaodan Shi, Yaxin Di, Zhiying Jia

**Affiliations:** 1 Heilongjiang River Fisheries Research Institute, Chinese Academy of Fishery Sciences, Harbin 150076, China; jiangxiaona@hrfri.ac.cn (X.J.);; 2 Key Laboratory of Freshwater Aquatic Biotechnology and Breeding, Ministry of Agriculture and Rural Affairs, Harbin 150076, China

**Keywords:** CyHV-3, carp strains and hybrids, viral gene expression, immune-related enzymes, immune-related genes

## Abstract

Cyprinid herpesvirus 3 (CyHV-3) is a pathogen that causes high mortality in common carp (*Cyprinus carpio*) and koi. Common carp breeding lines with different genetic backgrounds exhibit different resistance levels to viral pathogens. This study aimed to determine the differences in CyHV-3 disease resistance performance between the hybrid offspring (Y × M and M × Y) of the mirror carp ‘Longke 11’ (resistant to CyHV-3) and Yellow River carp, as well as the self-crossed offspring (M and Y). The M, Y × M, M × Y and Y groups were infected with CyHV-3 by immersion. The order of mortality and the duration of death for the four groups of carp were as follows: Y group > Y × M group > M × Y group > M group. Throughout the entire infection stage, the mRNA expression levels of the viral factors *thymidine kinase* (*TK*) and *open reading frame 72* (*ORF72*) in the four groups of carp tended to first increase but then decrease. The viral factor expression evaluated on days 30 and 31 post-infection (p.i.), which was the peak of infection mortality, was the highest in the Y group and the lowest in the M group, and compared with the Y × M group, the M × Y group had considerably lower viral gene expression (*p* < 0.05). The immune-related enzyme activity and content levels of the four carp groups matched the patterns of viral gene expression. On day 29 p.i., a time point with high mortality, the levels of alkaline phosphatase (AKP), glutathione peroxidase (GSH-Px) and total antioxidant capacity (T-AOC) were significantly the lowest in the Y group and significantly the highest in the M group, while the Y × M group showed a significant decrease compared to the M × Y group (*p* < 0.05). Quantitative real-time (q-PCR) analysis revealed that *interleukin-21 receptor* (*IL21R*), *interferon regulatory factor 9* (*IRF9*), *interferon type I* (*IFN-I*), interleukin-6 (*IL-6*) and *microtubule-associated protein light chain 3* (*LC3*), exhibited an initial increase followed by a decrease among the four experimental groups of common carp. In the peak mortality period of carp in the four groups (30 days post-infection), the expression levels of *IL21R*, *IRF9*, *LC3*, and *IFN-I* were significantly the highest in the M group and significantly the lowest in the Y group, with the mRNA expression of these genes in the M × Y group being significantly higher than that in the Y × M group (*p* < 0.05). In contrast, *IL-6* expression levels exhibited the opposite trend. In this study, the M group exhibited the greatest resistance to CyHV-3, followed by the M × Y group, whose resistance was greater than that of the Y × M group, with the Y group showing the lowest disease resistance. Our findings demonstrate that hybridization modulates resistance to CyHV-3. Furthermore, we identified conserved immune signatures common to both susceptible and resistant carp, including the activation of nonspecific immunity and the upregulation of immune-associated genes.

## 1. Introduction

CyHV-3 was first reported in Israel in 1988 [1]. CyHV-3 is a pathogen that causes koi herpesvirus disease (KHVD) in carp, koi, and their variants, with high contagiousness and a mortality rate as high as 90–100% [2], causing severe economic losses to carp aquaculture worldwide [3]. However, the breeding of disease-resistant varieties or hybrids can fundamentally solve the problem of large-scale mortality caused by viral infections.

Common carp, a freshwater fish species characterized by its global distribution and diverse and extensive history of domestication, constitutes a vital dietary staple for numerous developing nations with large populations. In terms of global aquaculture production, the common carp is the third most common freshwater species [4]. Recently, a novel variety of mirror carp that is resistant to CyHV-3, named mirror carp ‘Longke 11’, has been developed by employing a synergistic approach combining population-based mass selection and molecular marker-assisted selection (Variety Registration Number: GS-01-001-2022). Asian cyprinids exhibit significantly increased survival following viral challenge with CyHV-3 [5]. In addition, studies have shown that the survival of the Ropsha carp strain, a hybrid derived from the crossbreeding of Amur wild carp and European common carp, is superior following challenge with CyHV-3 [6,7,8,9]. In contrast, koi, which are hybrids of European and Asian carp, have a lower survival rate following CyHV-3 viral infection [7]. Genetic heterogeneity among distinct carp populations is correlated with their differential susceptibility to viral pathogens [6]. The increased survival rate of some fish populations may be attributed to a more robust immune response, which can effectively modulate the development of viral infections. Throughout the progression of CyHV-3 infection in common carp, the body produces corresponding immune responses, including the upregulation of immune genes and increases in the levels of immunological physiological and biochemical indicators [10,11,12]. In this study, the disease-resistant and fast-growing mirror carp ‘Longke 11’ and Yellow River carp were used as research objects. We evaluated the survival rates of the hybrid offspring and self-crossed offspring of the two carp species throughout natural CyHV-3 infection, as well as the temporal expression profiles of their viral genes (*TK* and *ORF72*). Changes in the levels of immune-related physiological and biochemical indicators AKP, GSH-Px, and T-AOC in hybrid and self-crossed offspring during different infection periods, as well as changes in the mRNA expression of the immune-related genes *IL-21R*, *IRF9*, *IFN-I*, *IL-6* and *LC3* were determined. This study revealed differential resistance to CyHV-3 in the self-crossed and hybrid offspring of the mirror carp ‘Longke 11’ and Yellow River carp, identified CyHV-3-resistant hybrid combinations, and laid the groundwork for the genetic selection of new disease-resistant strains.

## 2. Materials and Methods

### 2.1. Experimental Diets

The Yellow River carp and mirror carp ‘Longke 11’ were sourced from the Kuandian Fisheries Experimental Station, which is affiliated with the Heilongjiang River Fisheries Research Institute, Chinese Academy of Fishery Sciences. Additionally, the Kuandian Aquaculture Experimental Station has reported no cases of CyHV-3 infection in the past five years. The sexually mature mirror carp ‘Longke 11’ and Yellow River carp were self-crossed and hybridized to obtain the offspring of the mirror carp ‘Longke 11’ ♀ × Yellow River carp ♂ (M × Y), Yellow River carp ♀ × mirror carp ‘Longke 11’ ♂ (Y × M), mirror carp ‘Longke 11’ (M), and Yellow River carp (Y).

### 2.2. Infection Experiments

The experimental fish were newly hatched common carp in their first year of life. When the body length of the experimental fish (offspring fish) reached approximately 4–5 cm, indirect enzyme-linked immunosorbent assay (ELISA) was employed to confirm their seronegative status for CyHV-3 and any other herpesviruses. Four groups of common carp were placed into identical breeding water tanks (5 m × 6 m ×2 m), with 5000 individuals per water tank. Each group of common carp was placed into three tanks, with appropriate mesh dividers installed between the tanks to ensure water circulation among them. Prior to the challenge infection experiment, these experimental animals underwent an environmental acclimation period of more than 40 days, during which they were fed twice daily with commercial carp feed at a rate of 1.5% of the average body weight. We maintained the water temperature at 20 ± 1 °C and a pH of 7.0, which was continuously documented throughout the experiment. In accordance with the industry standard SC/T 7212.1-2011 [13], a PCR assay targeting the viral genes *TK* and *polymerase* (*Sph*) verified that all four carp groups were CyHV-3-negative (Figure S1a).

The fish infection experiment was performed following the protocol established by Jia et al. [14]. The infection method was achieved by cohabitation with CyHV-3-infected carp. Common carp exhibiting typical symptoms of CyHV-3 infection, which was molecularly confirmed by the PCR detection of *TK* and *Sph*, was utilized as the infection source. Subsequently, the PCR products were sequenced and compared with *TK* (GenBank: AB375390) and *Sph* (GenBank: AY568950). The homology rates with TK and Sph were 97.06% and 98.5%, respectively. According to the industry standard SC/T 7212.1-2011 [13], if the homology between the *TK* and *Sph* sequences is greater than 95%, the virus is determined to be CyHV-3. Additionally, the PCR primers used in this study for *TK* and *Sph* are consistent with those in previous studies [13,14,15]. These fish were donated by Dandong Yingbo Yalu River Ecological Technology Co., Ltd (Dandong, China). Infected fish were introduced into water tanks containing the four groups of carp at a ratio of 1:10 relative to the experimental fish population, and this method has been demonstrated to be feasible in the literature by Dixon et al. [8] and Jia et al. [14]. The water temperature during the infection period was 23 °C, a temperature shown to induce high mortality [16]. The daily mortality of the experimental fish was recorded. After 40 days post-experiment initiation, no further deaths occurred in the four groups of carp, and the experimental period lasted 50 days. Spleen and kidney tissues from the experimental fish were collected on days 7 (day of mortality onset), 26, 29, 30, 31, 32, 36, and 40 p.i. At least 15 fish were collected per group. All tissue samples were snap-frozen in liquid nitrogen and subsequently preserved at −80 °C. The PCR detection of the viral genes *TK* and *Sph* was performed to ensure that all the analyzed fish were infected with koi herpesvirus (Figure S1b).

### 2.3. DNA and RNA Extraction

Genomic deoxyribonucleic acid (DNA) was extracted from the spleen tissue using a DNeasy Tissue Kit (Qiagen, Shanghai, China). In accordance with the manufacturer’s instructions, total ribonucleic acid (RNA) was extracted from the spleen tissue using an RNeasy Mini Kit (Qiagen, Frankfurt, Germany). The integrity and quality of the DNA and RNA were assessed via 1.0% agarose gel electrophoresis, while their purity was quantified using ultraviolet radiation (UV) spectrophotometric analysis. All the RNA samples exhibited spectrophotometric purity, with OD 260:280 ratios ranging from 1.8 to 2.0, allowing for cDNA synthesis using a commercial reverse transcriptase kit (TaKaRa, Dalian, China).

### 2.4. q-PCR

In accordance with the instructions, the q-PCR experiment was conducted using TB Green™ Premix ExTaq™ II (TaKaRa, Beijing, China) and an ABI7500 system (Life Technologies, Carlsbad, CA, USA). The specificity of the primers was validated by analyzing the melting curve profiles (Table 1). Beta-actin (*β-actin*), previously validated as the optimal reference gene for mirror carp studies [15,17], served as the internal control and exhibited high expression stability across all the samples. The negative control contained double-distilled water instead of the template DNA. The relative mRNA expression levels of the seven target genes were determined using the 2^(−ΔΔCt)^ method. Each experimental group was established with at least three replicate samples, and the Tm value measurements included three replicates per experimental group. Each q-PCR experiment was performed with three biological replicates and four technical replicates.

### 2.5. Index Measurement

After static grinding, kidney tissue was diluted with physiological saline at a ratio of 1:9 and maintained in a low-temperature environment. Physiological and biochemical indicator levels were determined using an enzyme activity assay kit (Jiancheng, Nanjing, China). The activities of the total protein (TP, A045-2, Coomassie brilliant blue method), GSH-Px (A006-2-1, microplate method), and AKP (A059-2-2, microplate method) enzymes in kidney tissues were evaluated. Afterwards, 100 µL of ice-cold PBS solution was added to each 20 mg of tissue, and homogenization was performed to thoroughly disrupt the tissue and release the oxidants. The samples were subsequently centrifuged at 12,000× *g* for 5 min at 4 °C, after which the resulting supernatant was harvested for subsequent analysis. T-AOC was quantified using a commercial assay kit (S0116; Beyotime Institute of Biotechnology, Shanghai, China, FRAP method). Each experimental group comprised nine individuals (*n* = 9).

### 2.6. Data Statistics and Analysis

In this study, the survival rate was determined as the percentage of surviving fish relative to the total initial population in each group. The cumulative mortality rate is the ratio of the cumulative number of deaths in each period to the total initial population in each group. All the data were calculated and are reported as the mean ± standard deviation (SD) of three separate biological replicates. An one-way analysis of variance (ANOVA) was employed to assess the statistical significance of intergroup differences in the measured parameters. When significant differences were detected, multiple comparisons were performed using Duncan’s test. Statistical analysis was performed using IBM SPSS software (version 22.0; IBM Corp., Armonk, New York, NY, USA). Differences were considered statistically significant at *p* < 0.05 (* *p* < 0.05; ** *p* < 0.01; *** *p* < 0.001). The experiments consisted of at least three independent biological replicates.

## 3. Results

### 3.1. Survival Rate

**Figure 1:**
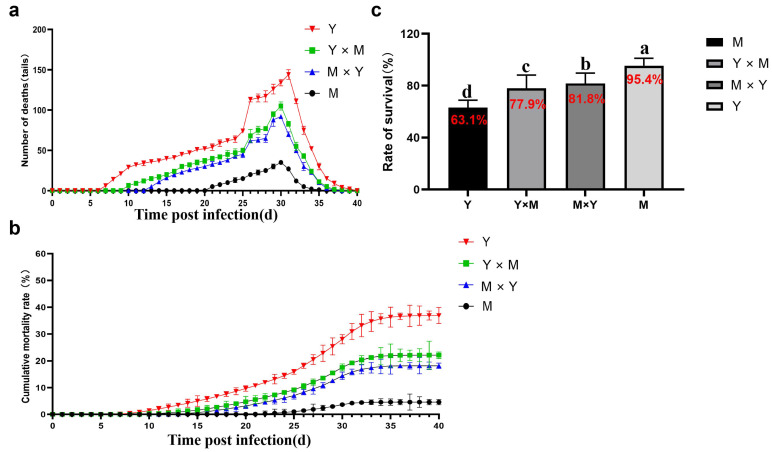
The number of dead fish, cumulative mortality rate and general survival rate among the four groups of carp after CyHV-3 infection. (**a**) A comparison of daily mortality among the four groups. Mortality was monitored immediately after viral challenge. *n* = 5000 per group. (**b**) The cumulative mortality rate of the four groups after CyHV-3 challenge (*n* = 5000 per group). (**c**) The general survival rates of the four groups. The data are shown as the means ± SDs of *n* = 5000 fish. Different lowercase letters indicate significant differences between two infection periods (*p* < 0.05).

The mortality of the four groups of carp was determined throughout the progression of CyHV-3 infection (Figure 1). The results revealed that fish mortality began in the Y group on day 7 p.i. and stopped on day 39 p.i., with a total of 1845 dead fish, resulting in a survival rate of 63.10%. The Y × M group began to die on day 10 p.i. and stopped dying on day 38 p.i., with a total of 1105 fish deaths and a survival rate of 77.90%. The M × Y group began to die on day 13 p.i. and stopped dying on day 37 p.i., resulting in a total of 910 fish deaths and a survival rate of 81.8%. However, the fish in the M group began to die on day 21 p.i. and stopped dying on day 35 p.i., with 230 fish deaths and a survival rate of 95.4%. There was a significant difference in mortality rates among the four groups of fish, with group M having the highest and group Y having the lowest (*p* < 0.05). Interestingly, the Y group reached peak mortality on day 31 p.i., whereas the mortality peak for the remaining three groups of carp occurred on day 30 p.i.

### 3.2. Relative Expression of Viral Genes

**Figure 2:**
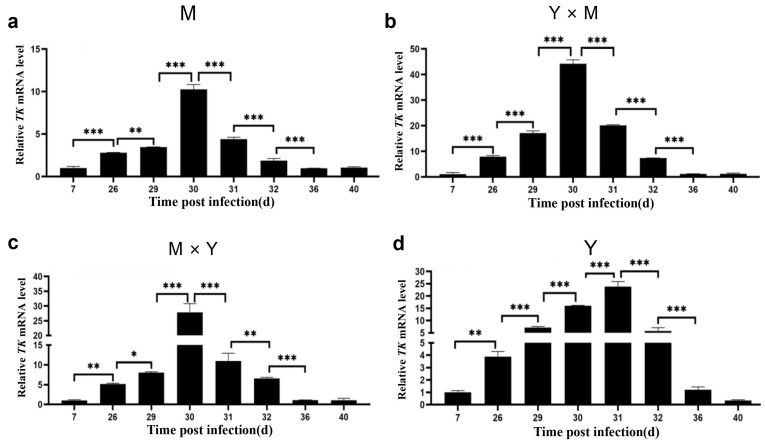
The relative expression levels of *TK* in the four groups of common carp spleen tissues during the eight stages of CyHV-3 infection. (**a**) M group; (**b**) Y × M group; (**c**) M × Y group; (**d**) Y group. The mRNA expression level of *TK* on day 7 p.i. was considered to be 1. The data are shown as the means ± SDs of *n* = 6 fish. Differences were considered statistically significant at *p* < 0.05 (* *p* < 0.05, ** *p* < 0.01 and *** *p* < 0.001).

**Figure 3:**
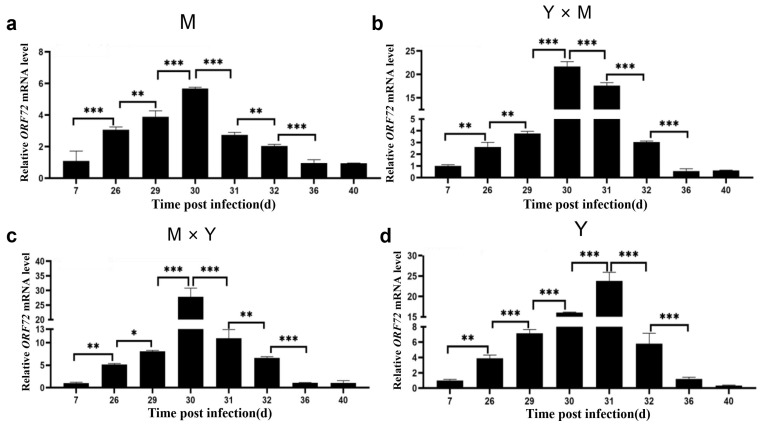
The relative expression levels of ORF72 in the four groups of common carp spleen tissues during the eight stages of CyHV-3 infection. (**a**) M group; (**b**) Y × M group; (**c**) M × Y group; (**d**) Y group. The mRNA expression level of TK on day 7 p.i. was considered to be 1. The data are shown as the means ± SDs of *n* = 6 fish. Differences were considered statistically significant at *p* < 0.05 (* *p* < 0.05, ** *p* < 0.01 and *** *p* < 0.001).

**Figure 4:**
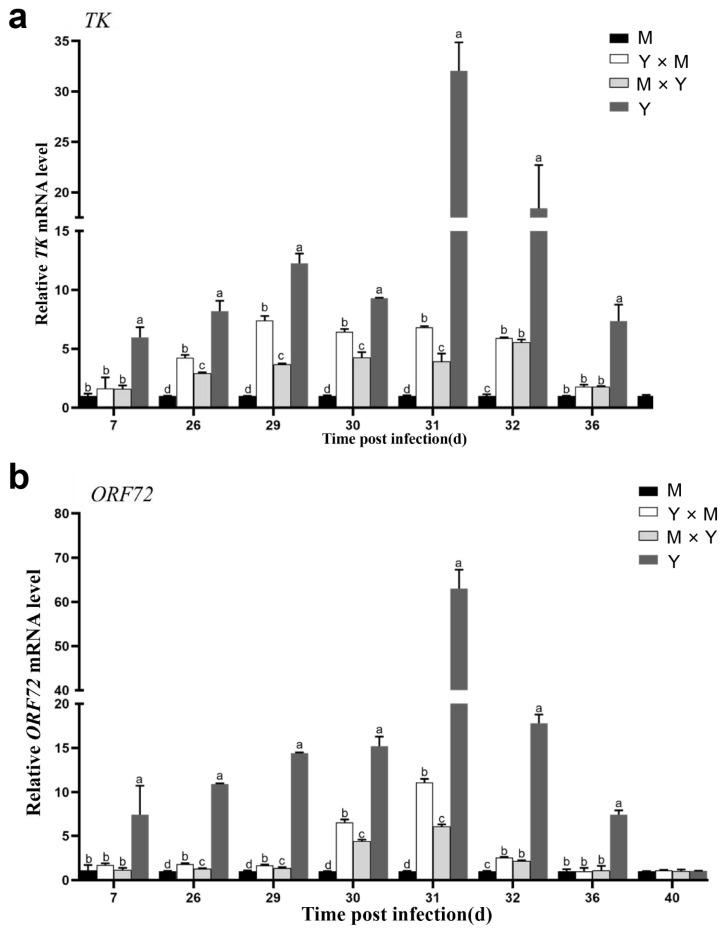
The relative expression levels of *TK* and *ORF72* in the four groups of common carp spleen tissues during the eight stages of CyHV-3 infection. (**a**) *TK*; (**b**) *ORF72*. In each infection stage, the *TK* or *ORF72* mRNA expression level of the M group was considered to be 1. The data are shown as the means ± SDs of *n* = 6 fish. Different lowercase letters indicate significant differences between two infection periods (*p* < 0.05).

The transcript levels of *TK* and *ORF72* in the spleen tissues of different groups of carp at different infection stages were determined. As shown in Figure 2 and Figure 3, the mRNA expression of *TK* and *ORF72* in the four groups of carp displayed similar trends, characterized by an initial increase followed by a decrease as the infection progressed. In the M, Y × M, and M × Y groups, the mRNA expression of *TK* and *ORF72* exhibited consistent expression patterns across different infection stages, with both being significantly greater on day 30 p.i. than at other stages (*p* < 0.001). Moreover, the relative expression levels of *TK* and *ORF72* in the Y group exhibited consistent expression patterns across different infection stages, with significantly higher levels on day 31 p.i. than at other infection stages (*p* < 0.001). In addition, except on day 40 p.i., the relative expression levels of *TK* and *ORF72* in group Y were significantly greater than those in the other three carp groups (*p* < 0.05) (Figure 4). Between days 26 and 32 p.i., the relative expression levels of *TK* and *ORF72* in the Y group were consistently the highest, while those in the M group were the lowest, across the four experimental groups (*p* < 0.05). From days 26 to 31 p.i., the relative expression levels of *TK* and *ORF72* in the Y × M group were significantly greater than those in the M × Y group (*p* < 0.05).

### 3.3. Immune-Related Enzyme Activity

**Figure 5:**
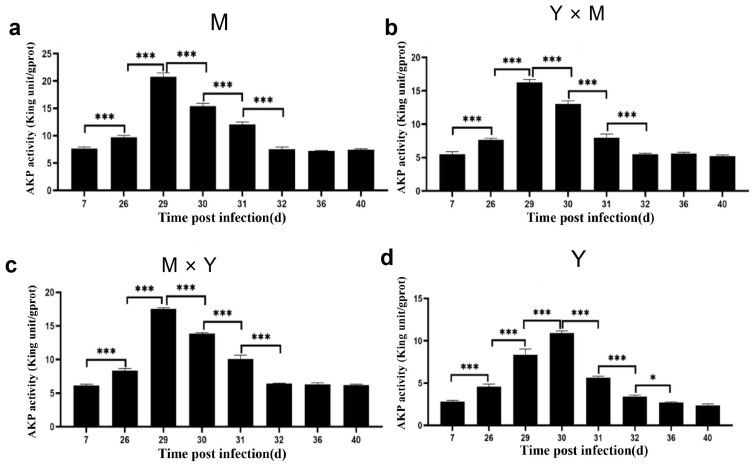
AKP activity in kidney tissues from the four groups of common carp during the eight stages of CyHV-3 infection. (**a**) M group; (**b**) Y × M group; (**c**) M × Y group; (**d**) Y group. The data are shown as the means ± SDs of *n* = 9 fish. Differences were considered statistically significant at *p* < 0.05 (* *p* < 0.05, and *** *p* < 0.001).

**Figure 6:**
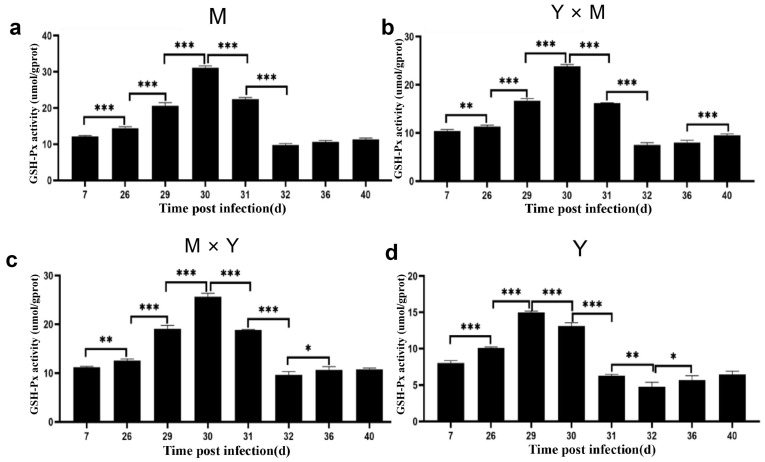
GSH-Px activity in kidney tissues from the four groups of common carp during the eight stages of CyHV-3 infection. (**a**) M group; (**b**) Y × M group; (**c**) M × Y group; (**d**) Y group. The data are shown as the means ± SDs of *n* = 9 fish. Differences were considered statistically significant at *p* < 0.05 (* *p* < 0.05, ** *p* < 0.01 and *** *p* < 0.001).

**Figure 7:**
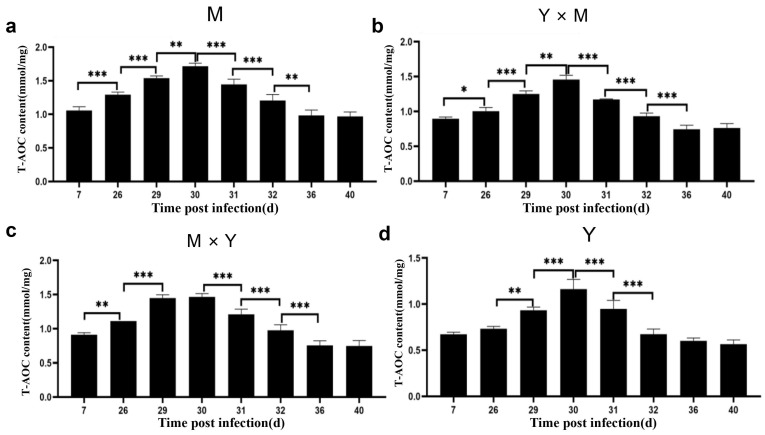
T-AOC activity in kidney tissues from the four groups of common carp during the eight stages of CyHV-3 infection. (**a**) M group; (**b**) Y × M group; (**c**) M × Y group; (**d**) Y group. The data are shown as the means ± SDs of *n* = 9 fish. Differences were considered statistically significant at *p* < 0.05 (* *p* < 0.05, ** *p* < 0.01 and *** *p* < 0.001).

**Figure 8:**
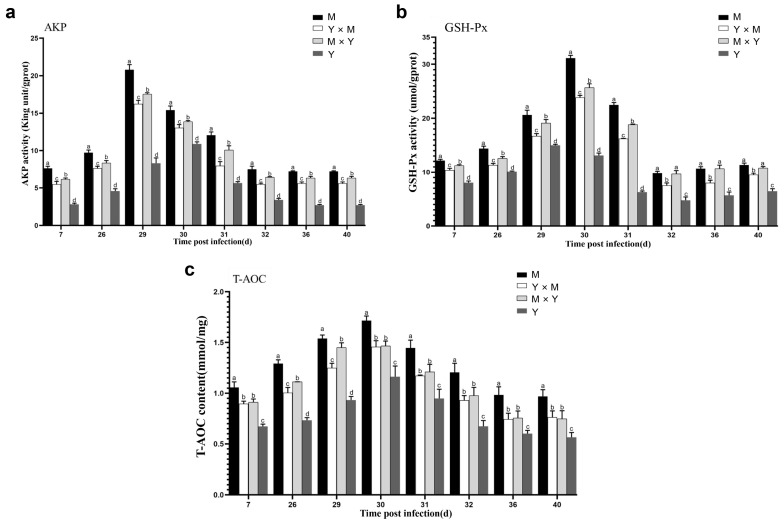
AKP activity, GSH-Px activity and T-AOC content in kidney tissues from the four groups of common carp during the eight stages of CyHV-3 infection. (**a**) AKP activity; (**b**) GSH-Px activity; (**c**) T-AOC content. The data are shown as the means ± SDs of *n* = 9 fish. Different lowercase letters indicate significant differences between two infection periods (*p* < 0.05).

The activities of the AKP and GSH-Px enzymes in the kidney tissues of the four groups of carp at different infection stages were determined. As shown in Figure 5, Figure 6 and Figure 7, with the progression of infection, the activity of common carp AKP and T-AOC in the four groups tended to first increase but then decrease, whereas the activity of GSH-Px tended to first increase, then decrease, and finally increase again. In the M group and Y × M group, AKP activity was significantly greater on day 29 p.i., while both GSH-Px activity and T-AOC content were significantly greater than those during the other infection periods on day 30 p.i. (*p* < 0.001). Interestingly, compared with those during the other infection periods, AKP activity and T-AOC content were significantly greater on day 30 p.i., while GSH-Px activity was significantly greater on day 29 p.i. in the Y group (*p* < 0.001). Furthermore, at all infection stages, the activity of AKP, the content of GSH-Px, and the content of T-AOC were the lowest in group Y, whereas the activity of AKP and the content of T-AOC were the highest in group M (*p* < 0.05) (Figure 8). The AKP activity in the M × Y group was significantly greater than that in the Y × M group throughout the entire infection course (*p* < 0.05) (Figure 8a). From days 7 to 31 p.i., the activity of the GSH-Px enzyme was significantly greater in the M group and significantly greater in the M × Y group than in the Y × M group (*p* < 0.05). Between days 32 and 40 p.i., the GSH-Px activity in the M group and M × Y group was significantly greater than that in the remaining two groups (*p* < 0.05) (Figure 8b). On days 26 and 29 p.i., the T-AOC levels in the M × Y group were significantly greater than those in the Y × M group (*p* < 0.05) (Figure 8c).

### 3.4. Expression of Immune-Related Genes

**Figure 9:**
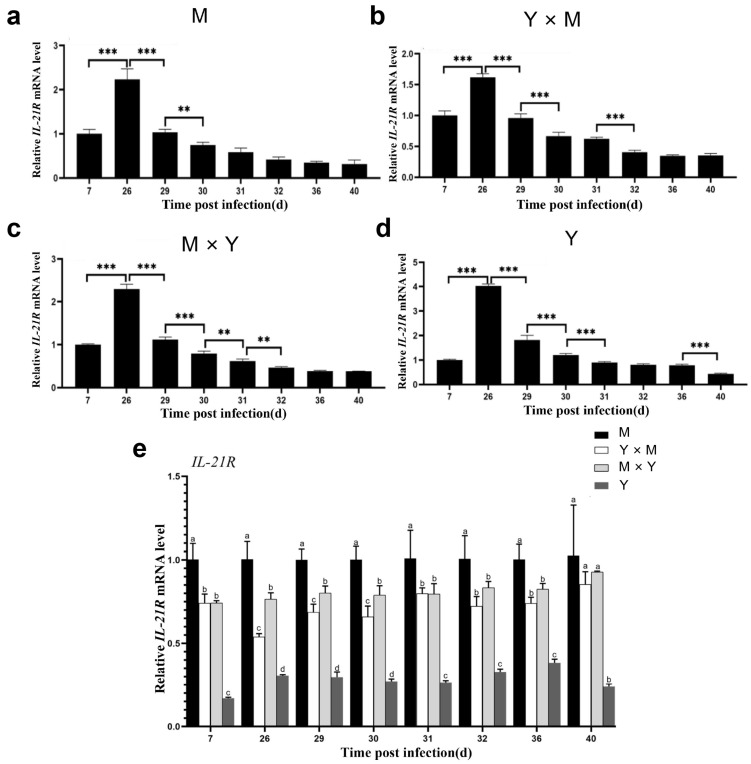
Relative expression levels of *IL21R* in four groups of common carp spleen tissues during eight stages of CyHV-3 infection. Relative mRNA expression of *IL21R* compared with that on day 7 p.i. (**a**–**d**) and in M group (**e**). Data are shown as means ± SDs of *n* = 6 fish. Differences were considered statistically significant at *p* < 0.05 (** *p* < 0.01 and *** *p* < 0.001). Different lowercase letters indicate significant differences between two infection periods (*p* < 0.05).

**Figure 10:**
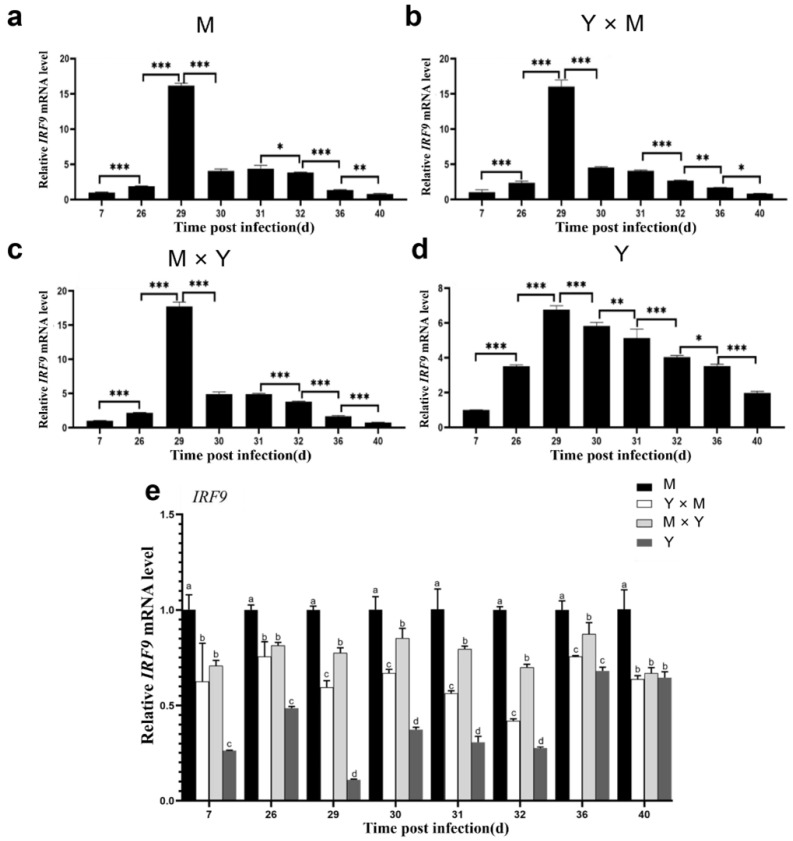
The relative expression levels of *IRF9* in the four groups of common carp spleen tissues during the eight stages of CyHV-3 infection. The relative mRNA expression of *IRF9* compared with that on day 7 p.i. (**a**–**d**) and in the M group (**e**). The data are shown as the means ± SDs of *n* = 6 fish. Differences were considered statistically significant at *p* < 0.05 (* *p* < 0.05, ** *p* < 0.01 and *** *p* < 0.001). Different lowercase letters indicate significant differences between two infection periods (*p* < 0.05).

**Figure 11:**
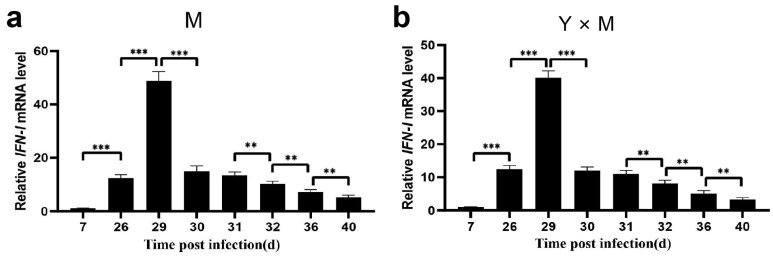

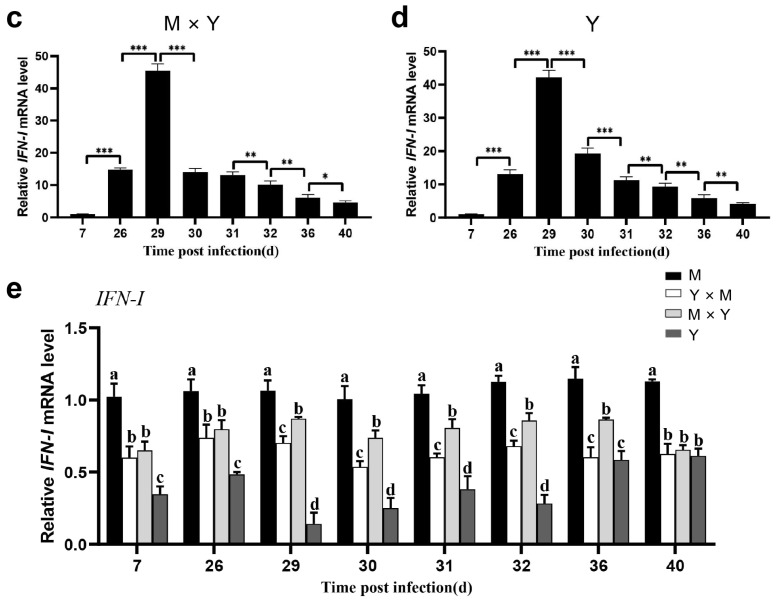
The relative expression levels of *IFN-I* in the four groups of common carp spleen tissues during the eight stages of CyHV-3 infection. The relative mRNA expression of *IFN-I* compared with that on day 7 p.i. (**a**–**d**) and in the M group (**e**). The data are shown as the means ± SDs of *n* = 6 fish. Differences were considered statistically significant at *p* < 0.05 (* *p* < 0.05, ** *p* < 0.01 and *** *p* < 0.001). Different lowercase letters indicate significant differences between two infection periods (*p* < 0.05).

**Figure 12:**
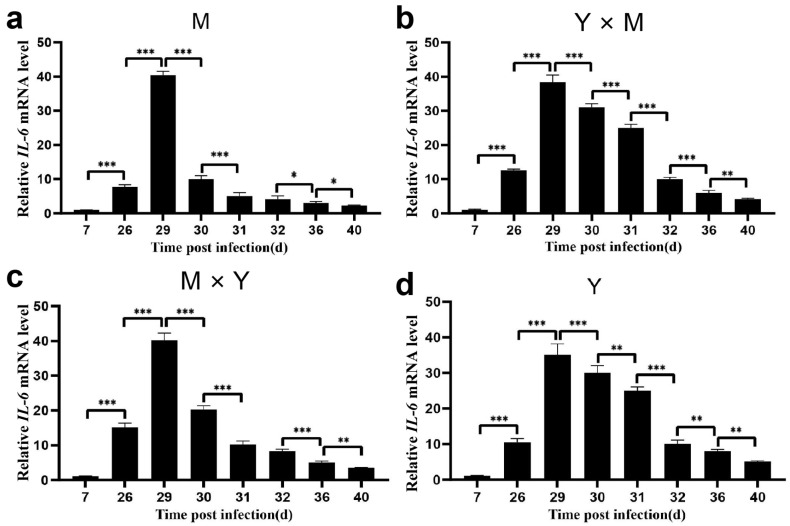

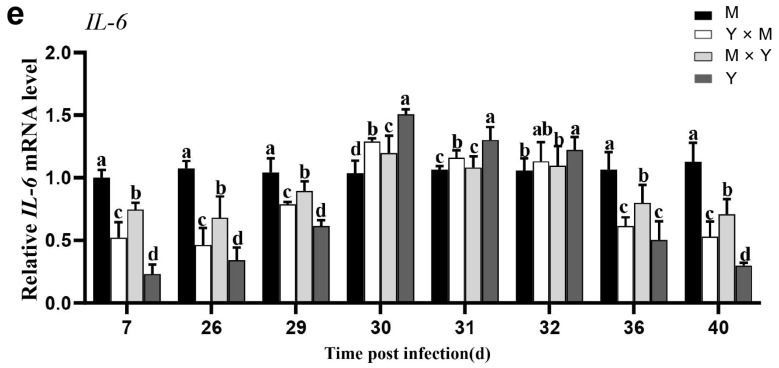
The relative expression levels of *IL-6* in the four groups of common carp spleen tissues during the eight stages of CyHV-3 infection. The relative mRNA expression of *IL-6* compared with that on day 7 p.i. (**a**–**d**) and in the M group (**e**). The data are shown as the means ± SDs of *n* = 6 fish. Differences were considered statistically significant at *p* < 0.05 (* *p* < 0.05, ** *p* < 0.01 and *** *p* < 0.001). Different lowercase letters indicate significant differences between two infection periods (*p* < 0.05).

**Figure 13:**
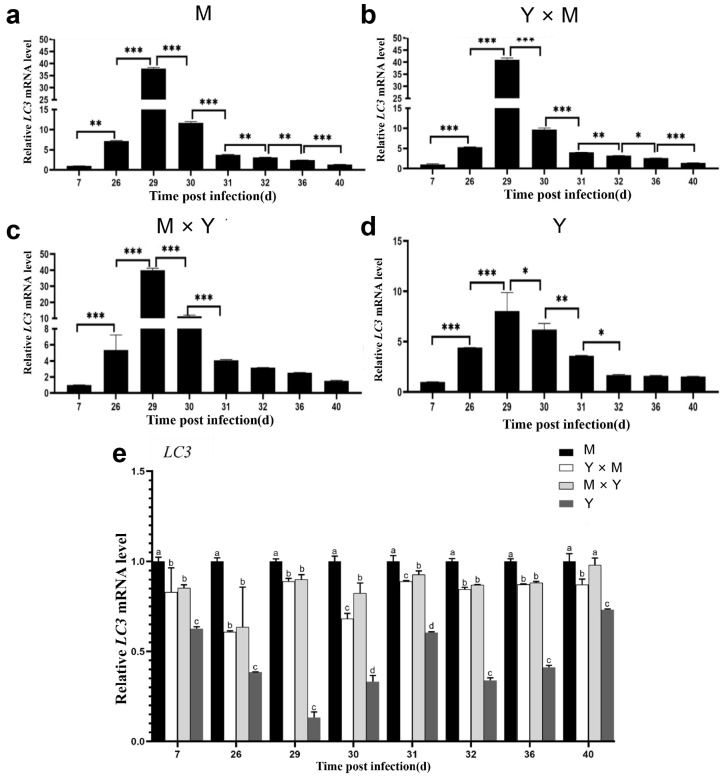
The relative expression levels of *LC3* in the four groups of common carp spleen tissues during the eight stages of CyHV-3 infection. The relative mRNA expression of *LC3* compared with that on day 7 p.i. (**a**–**d**) and in the M group (**e**). The data are shown as the means ± SDs of *n* = 6 fish. Differences were considered statistically significant at *p* < 0.05 (* *p* < 0.05, ** *p* < 0.01 and *** *p* < 0.001). Different lowercase letters indicate significant differences between two infection periods (*p* < 0.05).

The relative expression of *IL21R*, *IRF9*, *IFN-I*, *IL-6* and *LC3* was assessed in the spleen tissues of the four common carp groups across various infection stages. As the infection time gradually increased, the relative expression levels of *IL21R*, *IRF9*, *IFN-I*, *IL-6* and *LC3* in the four groups of carp tended to first increase but then decrease (Figure 9, Figure 10, Figure 11, Figure 12 and Figure 13). Compared with that at the other infection stages, the expression level of *IL21R* was significantly greater on day 26 p.i., while the expression of *IRF9*, *IFN-I*, *IL-6* and *LC3* was significantly greater on day 29 p.i. (*p* < 0.05). During the eight infection periods, the relative expression level of *IL21R* was significantly the lowest in the Y group and significantly the highest in the M group, except on day 40 p.i. (*p* < 0.05). Compared with the Y × M group, the M × Y group exhibited significantly elevated *IL21R* mRNA expression on days 26, 29, and 30 p.i. (*p* < 0.05) (Figure 9e). During different stages of infection, the relative expression levels of both *IRF9* and *IFN-I* in the M group were significantly greater than those in the other groups (*p* < 0.05). Except on days 36 and 40 p.i., the mRNA expression levels of *IRF9* and *IFN-I* in the Y group were significantly lower than those in all the other experimental groups (*p* < 0.05). On days 29, 30, 31, 32, and 36 p.i., the relative expression levels of *IRF9* and *IFN-I* in the M × Y group were significantly greater than those in the Y × M group (*p* < 0.05) (Figure 10e and Figure 11e). Furthermore, on days 7, 26, 29 and 40 p.i., the relative expression level of *IL-6* was significantly the highest in the M group, notably higher in the M × Y group than in the Y × M group, and significantly the lowest in the Y group (*p* < 0.05). Interestingly, on days 30 and 31 p.i., the expression level of *IL-6* mRNA in the Y group was significantly higher than that in all other experimental groups (*p* < 0.05) (Figure 12e). Except on day 40 p.i., the mRNA expression of *LC3* was significantly the lowest in the Y group and significantly the highest in the M group among all the experimental groups (*p* < 0.05). The expression levels of *LC3* in the M × Y group were significantly greater than those in the Y × M group on days 30 and 31 p.i. (*p* < 0.05) (Figure 13e).

## 4. Discussion

Breeding animals with different genetic backgrounds in targeted and nontargeted breeding programs can result in the development of offspring that are more resistant to disease [5]. In this study, the disease resistance of the hybrid and self-hybridized offspring of two CyHV-3-resistant carp strains was studied. During the CyHV-3 infection process, the order of mortality and the death duration of the four groups of carp from high to low was Y, Y × M, M × Y, and M, indicating that the M group was the strongest, group Y was the weakest, groups Y × M and M × Y were at an intermediate level, and group M × Y was stronger than group Y × M in terms of its ability to resist CyHV-3 infection. Common carp strains have stronger disease resistance to CyHV-3, and their body recovers faster after infection [18]. The results revealed that mortality ceased in the four groups in the following order: M, M × Y, Y × M, and Y. Significant differences in survival rates were observed among the four carp groups following CyHV-3 infection, with the disparity between the M and Y groups being the most pronounced. These results revealed differences in the survival rates of different carp strains after infection with CyHV-3, which was similar to the results of previous reports [7,19]. Studies have shown that *TK* and *ORF72* are involved in deoxynucleotide triphosphate synthesis during CyHV-3 replication and that their expression levels are positively correlated with the degree of viral replication [15,20]. The results of this study revealed that the relative expression of *TK* and *ORF72* in the four carp groups peaked but then decreased throughout the infection stage. This trend was consistent with previous reports on CyHV-3 gene expression [21]. Furthermore, this decline suggested that the host immune response gradually intensifies to suppress viral proliferation, which aligns with the mortality trends observed at different infection stages [5]. These findings further indicate that the relative expression of *TK* and *ORF72* in common carp could be used as indicators to evaluate host antiviral ability. In addition, the relative expression of *TK* and *ORF72* in the Y group was significantly greater than that in the other three groups of carp throughout the infection stage (*p* < 0.05), and this group exhibited the greatest death duration. On the basis of the above results, the immune response of the Yellow River carp after virus infection may have been weaker than that of the other three groups of carp, further verifying that the anti-CyHV-3 disease performance of the Yellow River carp was weaker than that of the other three groups of carp. On the basis of the above results, hybridization can alter the sensitivity of carp to CyHV-3 infection, which has been confirmed in the breeding of carp and crossbreeds in the Czech Republic [7]. Additionally, viral gene expression corresponding to immune responses during the infection period in group M has been reported multiple times, which is similar to the results of this study [13,14,21]. However, there are no reports on the differences in susceptibility to CyHV-3 between the offspring of the M group and other carp after hybridization. This study also revealed that the sensitivity of the two hybrid groups to CyHV-3 infection differed, with the survival rate of the M × Y group being greater than that of the Y × M group. When fish are infected with multiple viruses, the intensity of the immune response plays a key role in determining disease outcome. This phenomenon is the most evident in rainbow trout infected with viral haemorrhagic septicemia virus (VHSV) [22], and studies on Prussian carp infected with CyHV-3 virus have confirmed this phenomenon [23]. Like those in mammals, immune memory and antibody-mediated immune responses play crucial roles in the protective mechanisms against infections by various pathogens in fish [24].

As an important detoxification enzyme and immune reaction enzyme for the host, AKP participates in immune activities through nonspecific immunity and in the killing and digestion of pathogens in the body [25,26]. Studies have shown that during bacterial or viral infections, the activity level of AKP is significantly elevated in pompano (*Trachinotus ovatus*) and silver pomfret (*Pampus argenteus*) [27,28]. In this study, during the infection process, the AKP activity of the four groups of carp tended to first increase but then decrease. The AKP activity in the M group, Y × M group, and M × Y group reached the maximum levels on day 29 p.i., whereas in the Y group, it reached the maximum levels on day 30 p.i. (*p* < 0.001). Consistent with the findings of this study, the AKP enzyme activity levels in common carp and rainbow trout (*Oncorhynchus mykiss*) also tended to significantly increase, followed by a decrease after bacterial or viral infection [26,29], suggesting that CyHV-3 infection might disrupt the innate immunity of carp through viral proliferation [30,31]. Antioxidant reactions are important defence mechanisms for reducing intracellular oxidative stress levels [32,33]. The increased activity of the antioxidant enzyme GSH-Px results in the scavenging of free radicals generated by pathogen invasion, thereby enhancing host disease resistance. T-AOC is a comprehensive indicator that can be used to evaluate the antioxidant status of the immune defence system [34]. During the early stage of infection, both GSH-Px activity and T-AOC content significantly increased, indicating that the four types of carp generated strong antioxidant capacity during the initial phase of the antiviral response. In addition, the duration of increased GSH-Px activity varied. In the Y group, GSH-Px activity was the highest on day 29 p.i. (*p* < 0.001), whereas in the other three groups of carp, it was the highest on day 30 p.i. (*p* < 0.001). Furthermore, throughout the entire infection period, the GSH-Px activity in group Y was significantly lower than that in the other three groups (*p* < 0.05). However, the cumulative mortality in group Y was significantly greater than that in the other three groups on days 29 and 30 p.i., indicating that GSH-Px activity might play a crucial role in the immune response against CyHV-3. Furthermore, throughout infection, the activity of GSH-Px in the M and M × Y groups was significantly greater than that in the Y × M group (*p* < 0.05), suggesting that the M and M × Y groups possessed superior antioxidant capacity and enhanced defence against CyHV-3 infection and replication. These findings were consistent with the finding that compared with that in ordinary grass carp, the activity of GSH-Px in disease-resistant grass carp was greater [35]. The T-AOC content reached the highest level in all four carp species at 30 p.i. In the absence of infection, the T-AOC content was significantly the highest in the M group and significantly the lowest in the Y group (*p* < 0.05), suggesting that the M group might exhibit the greatest antioxidant capacity among the four groups. In largemouth bass (*Micropterus salmoides*), the protective effect against largemouth bass frog virus can be enhanced by increasing the T-AOC content and GSH-Px activity [36]. These results indicated that antioxidant capacity could play a pivotal role in the host immune response to CyHV-3 infection in carp.

CyHV-3 infection can induce complex immune responses in the host and trigger the differential expression of multiple immune molecules [2]. *IL21R* modulates the inflammatory response to CyHV-3 in common carp by inhibiting the STAT3 signalling pathway [16,37]. *IRF9* inhibits CyHV-3 replication in carp during infection [15]. The high expression of *IFN-I* can inhibit CyHV-3 replication [5,38]. In this study, the expression levels of *IL21R* and *IRF9* in the four groups initially increased but subsequently decreased throughout the infection period. The relative expression of *IL21R* was significantly greater on day 26 p.i., whereas compared with that at the other time points, the expression of *IRF9* and *IFN-I* peaked significantly on day 29 p.i. (*p* < 0.05). These results indicated that *IL21R* responds earlier than *IRF9* and *IFN-I* do during the immune response to CyHV-3 infection. *IL21R* is present on the surface of immune cells, such as B cells, T cells, and natural killer cells, and can participate in the host antiviral infection process by regulating multiple immune signalling pathways [39,40]. Moreover, *IRF9* inhibits viral replication by regulating the expression of *IFN-I*, which is involved in the defence against viral infection [19]. This study revealed similar expression patterns of *IRF9* and *IFN-I* during CyHV-3 infection, suggesting that *IRF9* might also regulate *IFN-I*-mediated viral replication suppression through molecular mechanisms in the four common carp. CyHV-3 infection in carp might first trigger the host’s inflammatory response and then regulate the interferon response to inhibit viral replication, which requires further investigation. In mammals, the *IL-6* cytokine family plays a crucial role in the immune system and exhibits both pro-inflammatory and anti-inflammatory functions. Studies have shown that *IL-6* mRNA is significantly upregulated on day 5 p.i. of CyHV-3 infection in common carp [41], which is consistent with the findings of this study. Furthermore, studies have found that increased *IL-6* mRNA expression can enhance the body’s antiviral immune response, but its overexpression will lead to chronic inflammation and trigger autoimmune diseases, thereby promoting viral replication [42,43]. The results of this study demonstrated that on days 31 and 32 p.i., which were the peak period of viral gene expression (peak period of viral gene expression), the *IL-6* mRNA expression level in the Y group was significantly higher than that in all other experimental groups, suggesting that the overexpression of the *IL-6* mRNA in the Y group promoted viral replication within the host. Previous studies have confirmed that *IL-6* can regulate inflammatory responses to facilitate CyHV-3 replication [44]. In addition, autophagy is the core mechanism that promotes the transport of various cellular contents to lysosomes for degradation and recycling [45]. Studies have shown that certain viral infections can alter autophagy levels and that autophagy can function as an antiviral pathway [46]. *LC3* is a key protein marker for phagosome formation and is widely used to detect autophagic activity [47]. As a defence mechanism, autophagy and the key protein *LC3* are involved in the later stages of CyHV-3 infection [48], and similar results have been reported for siniperca chuatsi rhabdovirus (SCRV) and singapore grouper iridovirus (SGIV) [49,50]. In this study, the relative expression of *LC3* in the four carp groups initially increased but then decreased, with significant differences observed among the time points (*p* < 0.05). This pattern indicated that CyHV-3 infection induces a dynamic autophagic response in the host, which is consistent with previous findings [48]. Except on days 36 and 40 p.i., the relative expression levels of *IL21R*, *IRF9*, *LC3* and *IFN-I* in the M group were significantly greater than those in the other three groups, whereas the levels in group Y were significantly lower (*p* < 0.05). The mortality data of the four groups of carp in the preliminary study suggested that the antiviral innate immune response triggered in the M group was superior to that triggered in the other three groups. Additionally, during the peak mortality period of the four groups of carp (30 p.i.), the expression of *IL21R*, *IRF9*, *LC3* and *IFN-I* in the M × Y group was significantly greater than that in the Y × M group (*p* < 0.05), However, the expression levels of *IL-6* showed the opposite trend. These results suggested that the M × Y group exhibited more intense immune responses triggered by immune factors and greater autophagy levels compared to the Y × M group, while the inflammatory response was weaker in the M × Y group. This might also be one of the reasons why the mortality rate in the M × Y group is lower than that in the Y × M group. Similar findings have been reported for various strains of common carp [41,48].

Interestingly, on the basis of comparative analyses of survival rates across different infection stages, viral replication dynamics, nonspecific immune responses, and the expression profiles of immune-related genes, resistance to CyHV-3 was significantly greater in the M × Y group than in the Y × M group. This phenomenon might be attributed to maternal effects or cytoplasmic inheritance, as supported by similar findings in other teleost species [51,52]. Studies have shown that different carp strains exhibit varying sensitivities to CyHV-3 and SVCV, with Ropsha carp demonstrating a high survival rate during both infections and Amur wild carp, Prerov scaly carp, and koi displaying a lower survival rate for CyHV-3 [5]. Moreover, intraspecies variability has a significant effect on the resistance level of a strain [6,9]. Further investigations are needed to determine whether the four types of common carp are susceptible to other viruses, such as SVCV, in this study. The results of this study confirm that hybridization can significantly increase the potential for disease resistance, achieving a successful case of disease resistance breeding. M × Y hybrids show promise for commercial application, pending further studies into their rearing and growth to promote the development of the common carp farming industry.

## 5. Conclusions

In summary, this study revealed significant differences in survival rates among the M, M × Y, Y × M, and Y groups during CyHV-3 infection. Furthermore, we characterized the differential expression of viral genes, immune response parameters, and immune-related factors across the four groups at various infection stages. The results of this study revealed that the M group exhibited the highest level of disease resistance. Compared with the other groups, the M group exhibited significantly higher survival rates, enhanced nonspecific immune responses, and elevated expression of immune-related factors during CyHV-3 infection, coupled with the lowest viral gene expression. Notably, the resistance of the M × Y hybrid was superior to that of the Y × M hybrid, whereas the resistance of the Y group was the poorest. This study identified shared features in the response to CyHV-3 infection between normal and resistant fish. These findings provide critical insights into the mechanisms of infectious disease resistance, fish immunogenetics, and the advancement of sustainable and safe aquaculture technologies.

## Figures and Tables

**Table 1:** All primer sequences in this study.

Primer	Sequences (5′-3′)	Tm	Application
*TK*	F: GGGTTACCTGTACGAG R: CACCCAGTAGATTATGC	58 °C	PCR
*Sph*	F: GGGTYACCTGTACGAG R: GACACATGTACAATGCTCGC	60 °C	PCR
*qTK*	F: CCCTTCACCGTCAGAATCTCTC R: AGCTCGTACTGGGCCATCC	60 °C	q-PCR
*qORF72*	F: CGAGAAGCAGGGTATGGTC R: GGCGTGTAGGGCACAAAG	60 °C	q-PCR
*qIL-21R*	F: TTGGGATGTGGGTTATACGGG R: GCCATCTTATCCGGGTCACT	62 °C	q-PCR
*qIRF9*	F: CCTACAAGGTGTACCGCCTCGTA R: ACTGTGTCTCCTTCTGCTGTTCCT	64 °C	q-PCR
*qLC3*	F: CCAATCAGGCTTTCTTCCTACTT R: TGTCTCCTGGGAGGCATAGAC	63 °C	q-PCR
*qIFN-I*	F: TCAGCTTTATTTTTCTTAGTGCCTT R: ACACGAGAGGAACATCTTTAGC	62 °C	q-PCR
*qIL-6*	F: CAAGTGTACCATCCCGAGCGTTG R: GCACAGAAGCGTCAGAGCAAGG	62 °C	q-PCR
*qβ-actin*	F: GCCGTGACCTGACTGACTACCT R: GCCACATAGAGAGCTTCTCCTTG	61 °C	q-PCR

## Data Availability

The original contributions presented in this study are included in the article/Supplementary Material. Further inquiries can be directed to the corresponding author(s).

## Supplementary Materials

The following supporting information can be downloaded at https://www.mdpi.com/article/10.3390/v18040432/s1, Figure S1: The agarose detection results of CyHV-3 infection in the four groups of carp. Before CyHV-3 infection (a) and after CyHV-3 infection (b). *TK*(A, B, C, D, E, F, G, H, I, J, K, L, M, N, O, P, Q, R, S), *Sph*(a,b,c,d,e,f,g,h,i,j,k,l,m,n,o,p,q,r,s); negative control (A, a); positive control (J, j) M group (B, b, C, c, D, d, E, e); Y × M group (F, f, G, g, H, h, I, i); M × Y group (K, k, L, l, N, n, O, o); Y group (P, p, Q, q, R, r, S, s).

## Abbreviations

The following abbreviations are used in this manuscript:

CyHV-3	cyprinid herpesvirus 3
TK	CyHV-3 thymidine kinase
Sph	CyHV-3 polymerase
ORF72	open reading frame 72
AKP	alkaline phosphatase
T-AOC	total antioxidant capacity
GSH-Px	glutathione peroxidase
q-PCR	quantitative real-time
IL21R	interleukin-21 receptor
IRF9	interferon regulatory factor 9
LC3	microtubule-associated protein light chain 3
IFN-I	interferon type I
IL-6	interleukin-6
KHVD	koi herpesvirus disease
M × Y	mirror carp ‘Longke 11’ ♀ × Yellow River carp ♂
Y × M	Yellow River carp ♀ × mirror carp ‘Longke 11’ ♂
M	mirror carp ‘Longke 11’
Y	Yellow River carp
DNA	deoxyribonucleic acid
RNA	ribonucleic acid
β-actin	beta-actin
TP	total protein
SD	standard deviation
ANOVA	one-way analysis of variance
SCRV	*Siniperca chuatsi* rhabdovirus
SGIV	Singapore grouper iridovirus
VHSV	viral haemorrhagic septicemia virus
ELISA	enzyme-linked immunosorbent assay
p.i.	post-infection
UV	ultraviolet radiation
