# Supplementary figures and images for: Differences in Susceptibility to Cyprinid Herpesvirus 3 (CyHV-3) Infection Among Carp (Cyprinus carpio L.) Strains and Hybrids
Source: Viruses. 2026 Apr 2;18(4):432. doi: 10.3390/v18040432 (PMC13119718; doi:10.3390/v18040432)

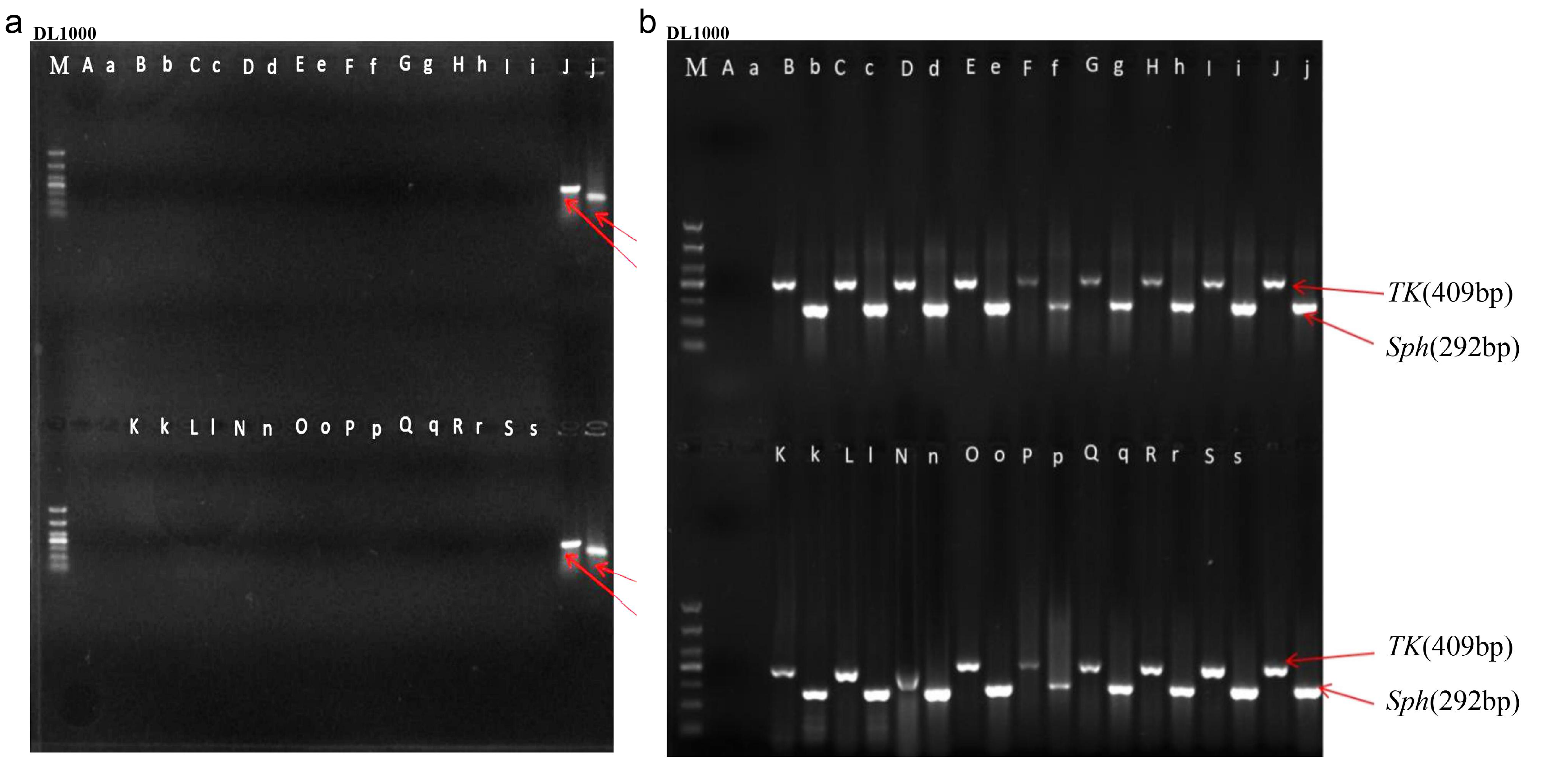

Supplement: Supplementary file 1 [file viruses-18-00432-s001.zip › viruses-4176088-Figure S1.tif]
